# A dynamic nomogram predicting symptomatic pneumonia in patients with lung cancer receiving thoracic radiation

**DOI:** 10.1186/s12890-024-02899-w

**Published:** 2024-02-26

**Authors:** Yawen Zha, Jingjing Zhang, Xinyu Yan, Chen Yang, Lei Wen, Minying Li

**Affiliations:** 1 Departments of Thoracic Cancer Radiotherapy, Zhongshan People’s Hospital, Zhanshan, China; 2 Xinxiang Medical University, Xinxiang, China; 3 Departments of Medical Oncology, Sun Yat-sen University Cancer Center, Guangzhou, China

**Keywords:** Dynamic nomogram, Lung cancer, Symptomatic pneumonia

## Abstract

**Purpose:**

The most common and potentially fatal side effect of thoracic radiation therapy is radiation pneumonitis (RP). Due to the lack of effective treatments, predicting radiation pneumonitis is crucial. This study aimed to develop a dynamic nomogram to accurately predict symptomatic pneumonitis (RP ≥ 2) following thoracic radiotherapy for lung cancer patients.

**Methods:**

Data from patients with pathologically diagnosed lung cancer at the Zhongshan People’s Hospital Department of Radiotherapy for Thoracic Cancer between January 2017 and June 2022 were retrospectively analyzed. Risk factors for radiation pneumonitis were identified through multivariate logistic regression analysis and utilized to construct a dynamic nomogram. The predictive performance of the nomogram was validated using a bootstrapped concordance index and calibration plots.

**Results:**

Age, smoking index, chemotherapy, and whole lung V5/MLD were identified as significant factors contributing to the accurate prediction of symptomatic pneumonitis. A dynamic nomogram for symptomatic pneumonitis was developed using these risk factors. The area under the curve was 0.89(95% confidence interval 0.83–0.95). The nomogram demonstrated a concordance index of 0.89(95% confidence interval 0.82–0.95) and was well calibrated. Furthermore, the threshold values for high- risk and low- risk were determined to be 154 using the receiver operating curve.

**Conclusions:**

The developed dynamic nomogram offers an accurate and convenient tool for clinical application in predicting the risk of symptomatic pneumonitis in patients with lung cancer undergoing thoracic radiation.

## Introduction

According to the latest global cancer statistics, lung cancer is the second most diagnosed cancer and the leading cause of cancer death, with an estimated 2.2 million new cancer cases and 1.8 million deaths [1]. Radiotherapy has been demonstrated to improve lung cancer survival rates and is widely used for treating various stages of lung cancer [2]. However, radiation pneumonitis (RP), as the most common and potentially fatal toxic side effect, there is no specific treatment for radiation pneumonia, which is mainly treated with corticosteroids, and failure of treatment can lead to death, which can offset the survival benefits of radiotherapy and impact survivors’ quality of life.^3–5^ Due to the lack of effective treatments for radiation pneumonitis, establishing an accurate RP prediction model is crucial [6].

Previous studies have shown that clinical factors such as older age, T stage, female, non-smokers, ECOG performance [3, 4], tumor location in the mid-lower lung, chemotherapy, targeted therapies, immune checkpoint inhibitors (ICIs), postoperative radiotherapy (PORT), total lung volume, smaller spared lung volume, and poor pulmonary function are closely related to RP incidence [3, 7–13]. Additionally, whole lung V5, V10, V20, mean lung dose (MLD), and normal tissue complication probability (NTCP) have been reported to be strongly associated with RP occurrence [14–16]. Theoretically, combining multiple risk factors can establish an accurate prediction model. Some researchers have successively developed prediction models combining clinical factors with dosimetric factors based on risk factors, with the highest AUC being 0.853 [17–21]. In recent years, radiomic analysis techniques have been widely used in studying treatment-related adverse reactions, and lung texture characteristics can be obtained using radiomic features, which can be utilized to describe potential RP risk [22, 23]. When combined with radiomic factors, the prediction performance was greatly improved, and the highest AUC could reach 0.94 [24–29]. However, radiomic features are difficult and complex to apply in clinical practice, particularly in primary hospitals.

The initial aim of this study was to validate the predictive performance of a symptomatic pneumonitis model based on lung dosimetry factors combined with clinical characteristics. Additionally, the optimal lung dose threshold was determined using this prediction model. More importantly, a dynamic nomogram was developed, which is a conveniently applicable prediction model integrating clinical and dosimetry characteristics to accurately predict the risk of developing symptomatic pneumonitis. Furthermore, the predictive power of this model is higher than previous models combining dosimetry factors with clinical characteristics.

## Materials and methods

A retrospective study was conducted on lung cancer patients who received radiation therapy at the Zhongshan People’s Hospital Department of Radiotherapy for Thoracic Cancer between January 2017 and June 2022. Inclusion criteria were: (1) patients with pathologically diagnosed stage I to stage III lung cancer (2), patients who received chest radiation therapy, and (3) no cause of death other than radiation pneumonitis within six months after radiotherapy. Exclusion criteria included: (1) patients who did not complete the entire radiotherapy course due to reasons other than radiation pneumonitis (2), incomplete follow-up data, and (3) radiotherapy interruption for more than one week. One hundred sixty patients were selected for this study based on the inclusion and exclusion criteria. Radiation pneumonitis was diagnosed and graded according to clinical symptoms, physical examination, and imaging evidence, following the commonadversereactioneventevaluationcriteria(CTCAE,5.0). Available: http://ctep.cancer.gov/protocolDevelopment/electronic_applications/ctc.htm.) The endpoint of this study was symptomatic pneumonitis, defined as RP grade two or higher occurring within six months of receiving radiation therapy.

Level 1: No symptoms, only clinical or imaging changes, and no treatment is required;

Level 2: Mild symptoms, limited work-related daily activities, need drug treatment;

Level 3: Severe symptoms, personal daily activities are limited, requiring oxygen inhalation;

Level 4: Life-threatening respiratory symptoms that require urgent treatment;

Level 5: Causing the death of the patient.

Enrolled patients were divided into symptomatic pneumonitis and asymptomatic pneumonitis groups, and independent prognostic factors were determined using univariate and multivariate Cox regression analyses. Based on the identified variables, the nomogram was developed and validated to predict symptomatic pneumonitis in patients with lung cancer receiving thoracic radiation. The discrimination, calibration, and clinical utility of the nomogram were evaluated using the concordance index (C-index), receiver operating characteristic(ROC) curve and the area under the curve (AUC), calibration curve, and decision curve analysis (DCA). Furthermore, a web-based survival calculator was developed based on the nomogram. Finally, each patient’s risk score was calculated to determine the optimal high and low-risk cut-off values.

### Statistical analysis

Univariate logistic regression analysis was used to identify potential risk factors for symptomatic pneumonitis. The chi-square test and t-test were employed to compare the baseline characteristics between the two groups. All statistical analyses were computed using the statistical software (SPSS 25.0). Factors with a *P*-value ≤ 0.10 in univariate analysis were included in further multivariate logistic regression analysis. Independent risk factors (OR>1and *P < 0.05 in multivariate logistic regression) were ultimately used to build the predictive model.* A nomogram was then constructed based on the independent risk factors of the multivariate logistic regression model using the “rms” and “DynNom” packages in the R project software (version 6.5). The regression model calculated a score for each variable, and the predicted probability of symptomatic pneumonitis could be derived by summing the scores of the individual variables. The receiver operating characteristic (ROC) curve with the value of the area under the curve (AUC) was further utilized to appraise the prediction efficiency of the model and calculate the optimal cut-off values of parameters. Calibration assessed the extent to which the predicted probabilities were consistent with the actual outcomes. Additionally, decision curve analysis (DCA) was used to evaluate the clinical utility of nomograms based on net returns to different probability thresholds. The receiver operating curve was employed to further determine the optimal cut-off values for high and low risk, as well as the cut-off values for Vx(percentage of whole lungs volume receiving x Gy) and MLD.

## Result

### Patient characteristics and incidence of symptomatic pneumonia (Grade ≥ 2)

**Fig. 1:**
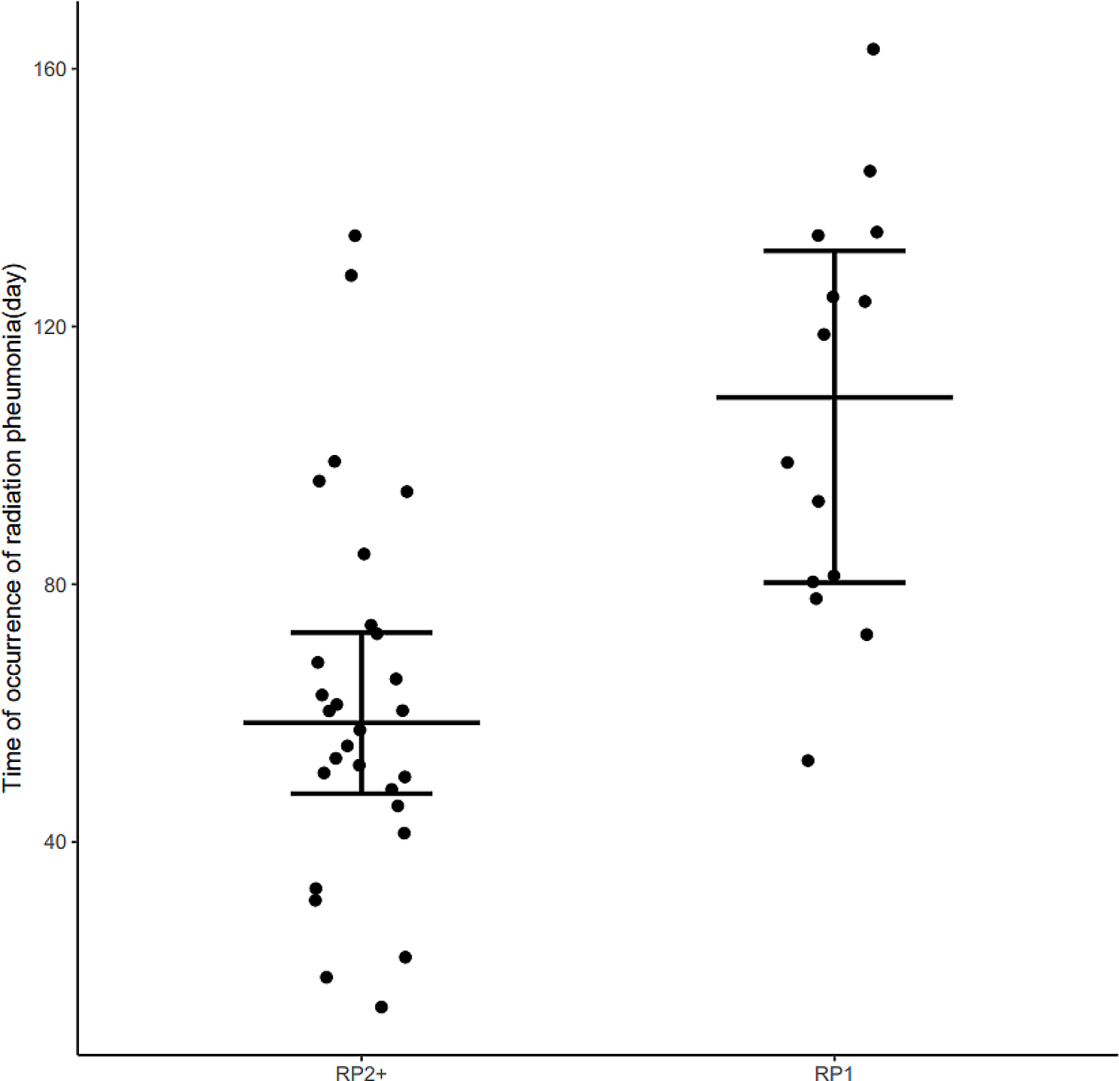
The occurrence time of radiation pneumonitis

The detailed characteristics of the enrolled population are presented in Tables 1 and 2. A total of 28 patients developed symptomatic pneumonitis, with the median interval from receiving the first radiation treatment to the appearance of symptomatic pneumonitis being 52 days (range, 14–134 days). The occurrence time of radiation pneumonitis is depicted in Fig. 1.

**Table 1:** Clinical characteristics of patients

Characteristic	All patients (*n*=160)	symptomatic pneumonia(*n*=28)	asymptomatic pneumonia (*n*=132)	univariate analysis		multivariate analysis	
				OR (95%CI)	*P*	OR (95%CI)	*P*
Gender				1.852(0.517–6.638)	0.344		
Male	133	25	108				
Female	27	3	24				
Age				0.181(0.065–0.505)	0.001	6.243(1.658–25.503)	0.007
≤ 60	77	5	72				
>60	83	23	60				
ECOG				1.022(0.353–2.961)	0.968		
≤ 1	131	23	108				
>2	29	5	24				
Smoking index				0.271(0.114–0.645)	0.003	4.498(1.306–15.489)	0.017
<400	93	9	84				
≥ 400	67	19	48				
Respiratory comorbidity				1.500(0.573–3.929)	0.409		
YES	31	7	24				
NO	129	21	108				
Tumor location				/	0.264		
Upper left lobe	33	3	30				
Lower left lobe	38	8	30				
Upper right lobe	47	8	39				
Lower right lobe (including middle lobe)	42	9	33				
Pathological types				/	0.488		
Adenocarcinoma	60	8	52				
squamous cell carcinoma	46	9	37				
Small cell carcinoma	52	10	42				
large cell carcinoma	2	1	1				
Stage (AJCC 8th)				/	0.215		
I	10	3	7				
II	18	4	14				
III	132	21	111				
IV	0	0	0				
Mutation of gene				0.558(0.121–2.576)	0.455		
YES	18	2	16				
NO	142	26	116				
Chemotherapy				0.222(0.094–0.527)	**0.001**	7.188(2.055–25.141)	0.002
YES	122	14	108				
NO	38	14	24				
Chemotherapy regimen				/	0.698		
Paclitaxel + cisplatin/carboplatin		2	31				
Pemetrexed + cisplatin/carboplatin		2	35				
Etoposide + cisplatin/carboplatin		10	42				
Immunotherapy				1.098(0.291–4.142)	0.890		
Yes	16	3	13				
NO	144	25	119				
Targeted therapy				1.098(0.291–4.142)	0.890		
Yes	16	3	13				
NO	144	25	119				
Postoperative Radiotherapy				1.149(0.393–3.362)	0.800		
Yes	26	5	21				
NO	134	23	111				
Pulmonary ventilation function				/	0.744		
Normal	42	9	33				
Mild abnormality	30	4	26				
Moderate abnormal	15	3	12				
Severe abnormality	13	3	10				
Extremely severe abnormality	3	0	3				
Radiotherapy mode				5.697(0.736–44.078)	0.177		
VMRT	136	27	109				
SBRT	24	2	22				
Dose fraction				/	0.247		
60 Gy/28-30 F	114	21	93				
45 Gy/30F	24	5	19				
50 Gy/25F	7	1	6				
40-60 Gy/8-10 F	7	1	6				
45-54 Gy/18F	2	0	2				
48-50 Gy/4-6 F	6	0	6				

**Table 2:** Dosimetry characteristics of patients

Characteristic	Symptomatic pneumonia(X ± S)	Asymptomatic pneumonia (X ± S)	Univariate analysis		Multivariate analysis	
			OR (95%CI)	*P*	OR (95%CI)	*P*
BED(Gy)	68.55 ± 11.87	71.89 ± 12.57	1.02(0.988–1.061)	0.198		
GTV (cm³)	56.28 ± 59.29	54.30 ± 74.03	1.000(0.995–1.006)	0.892		
GTVnd(cm³)	14.75 ± 18.42	12.69 ± 29.68	0.998(0.985–1.011)	0.726		
PTV (cm³)	353.82 ± 196.46	292.40 ± 173.68	0.998(0.996-1.000)	0.101	1.000(0.996–1.004)	0.881
Left lung volume(cm³)	1423.66 ± 343.58	1429.56 ± 436.85	1.000(0.999–1.001)	0.946		
Right lung volume(cm³)	1754.07 ± 468.70	1700.36 ± 491.30	1.000(0.999–1.001)	0.595		
Total Lung Volume(cm³)	3177.23 ± 729.26	3114.24 ± 866.33	1.000(0.999-1.000)	0.719		
V5 of total lungs(%)	64.98 ± 14.32	50.53 ± 18.42	0.945(0.916–0.975)	<0.001	1.083(1.012–1.159)	0.022
V10 of total lungs(%)	45.52 ± 12.04	38.34 ± 16.59	0.970(0.943–0.998)	0.035	0.855(0.747–0.978)	0.023
V20 of total lungs(%)	23.18 ± 8.67	18.00 ± 8.88	0.936(0.892–0.983)	0.008		
V30 of total lungs(%)	13.10 ± 6.31	9.59 ± 5.05	0.889(0.820–0.963)	0.004	1.17(0.634–2.159)	0.251
V40 of total lungs(%)	7.65 ± 4.34	5.62 ± 3.32	0.861(0.767–0.966)	0.011	0.752(0.423–1.336)	0.331
V50 of total lungs(%)	3.75 ± 3.41	2.98 ± 2.94	0.928(0.822–1.048)	0.231		
V60 of total lungs(%)	1.49 ± 1.15	1.02 ± 1.54	0.831(0.651–1.061)	0.137		
MLD of total lungs	1383.15 ± 324.92	1002.98 ± 505.05	0.998(0.997–0.999)	0.001	1.005(1.000-1.010)	0.005
MLD of ipsilateral lung	1793.29 ± 652.52	1464.68 ± 652.52	0.999(0.998-1.000)	0.011	1.00(0.999–1.002)	0.733
Max of total lungs	6195.95 ± 1117.16	5805.172 ± 1686.10	1.000(0.999-1.000)	0.256		
Min of total lungs(%)	53.13 ± 80.19	41.57 ± 107.76	0.999(0.996–1.002)	0.601		

### Univariate and multivariate analyses

The results of the univariate analysis are reported in Tables 1 and 2. Potential factors predicting RP ≥ 2 were identified as follows: age, smoking index, chemotherapy, PTV, lung V5, V10, V20, V30, V40, MLD of the total lung, and ipsilateral lung (all *P* ≤ 0.10). Multivariate analysis indicated that age, smoking index, chemotherapy, lung V5 and MLD of the whole lung were independent predictors of symptomatic pneumonitis (all *P < 0.05 and OR>1).* These factors were ultimately used to build our nomogram.

### Construction and validation of nomogram

**Fig. 2:**
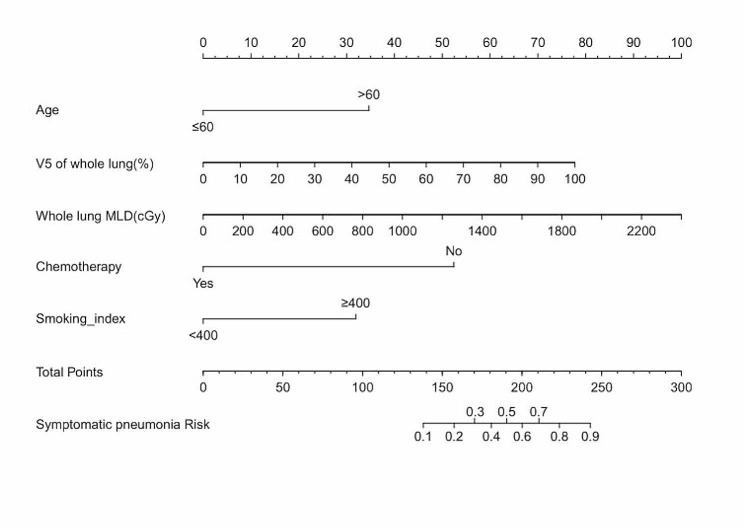
Predictive nomogram for symptomatic pneumonia

**Fig. 3:**
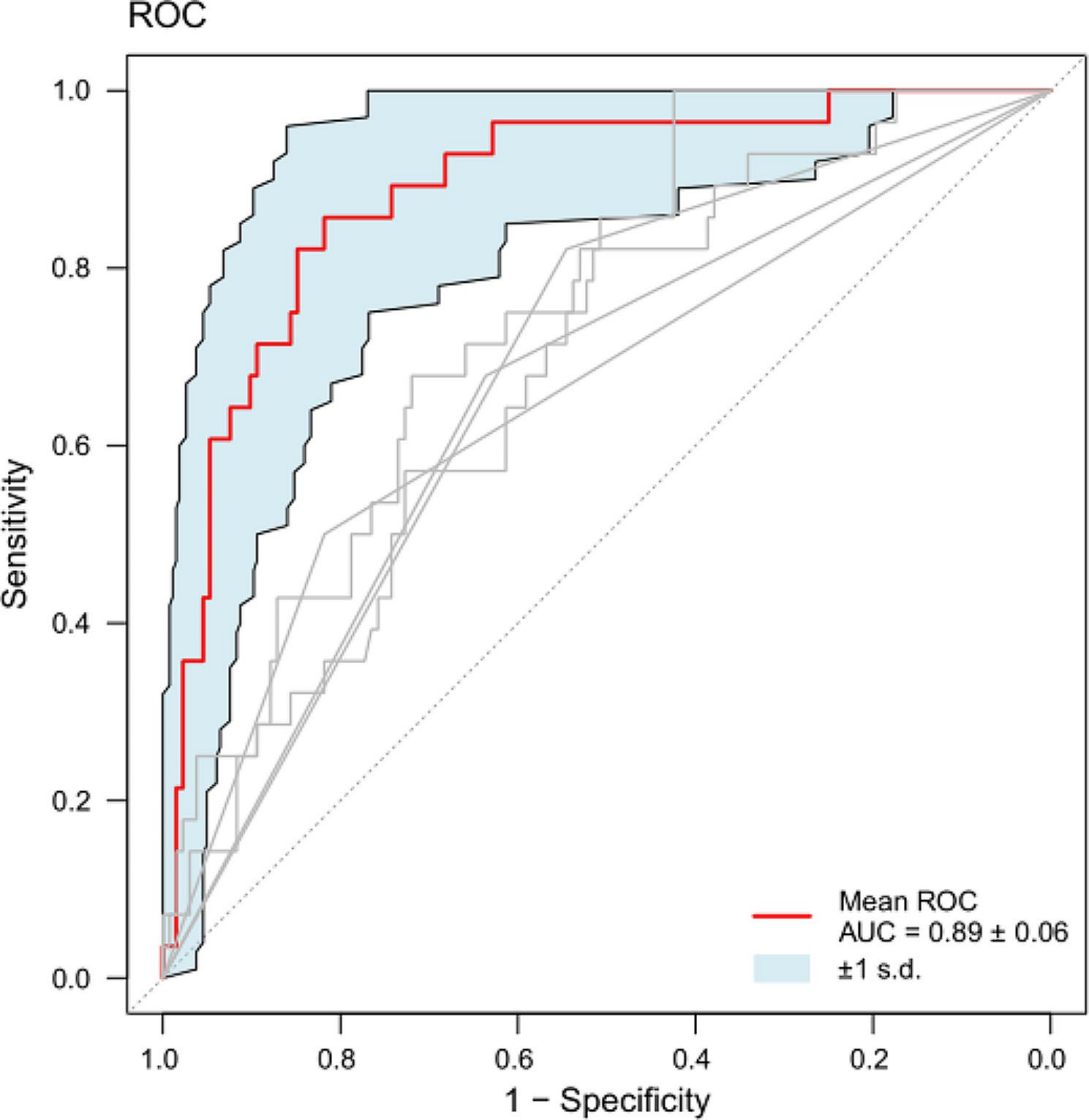
Receiver operating characteristic (ROC) curves

**Fig. 4:**
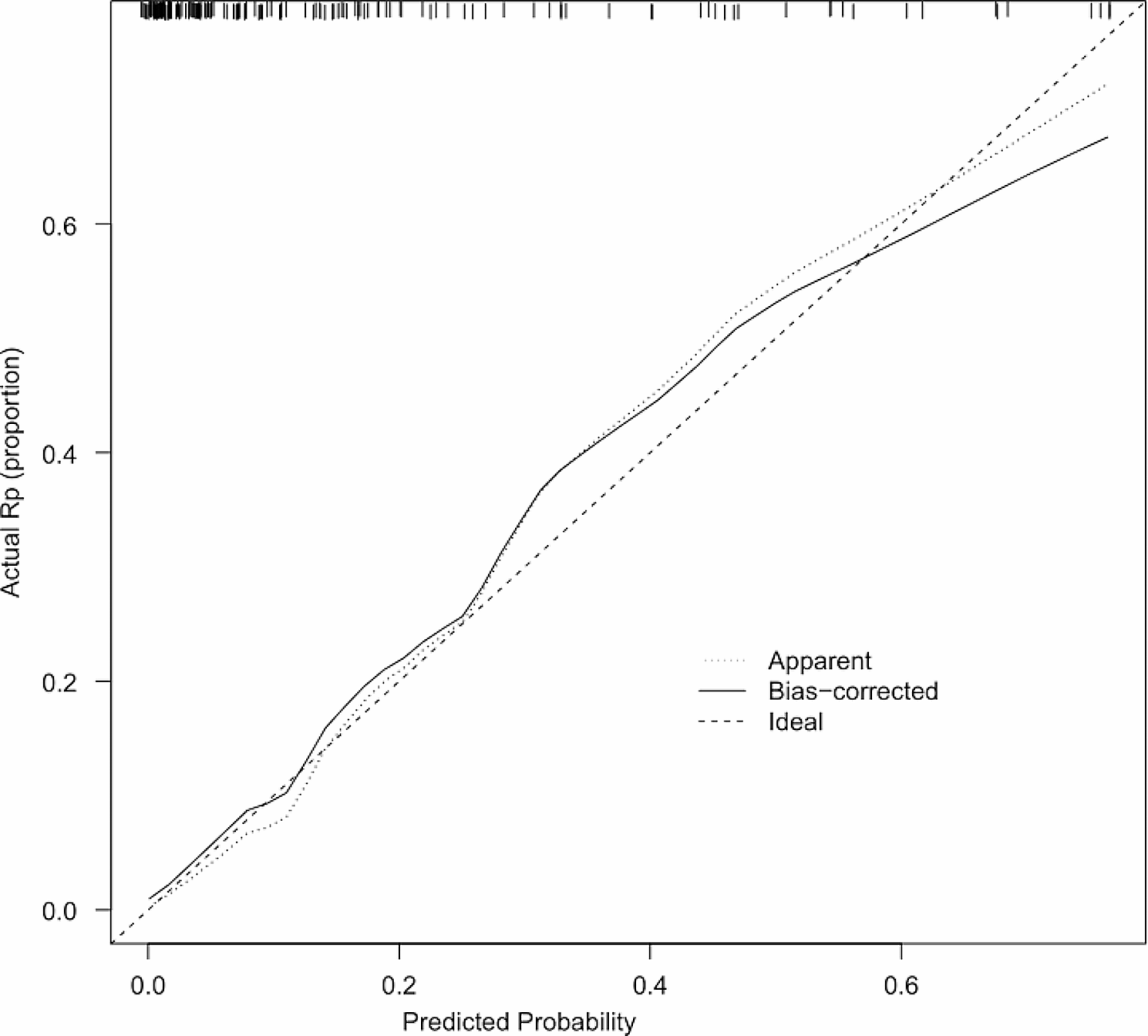
Calibration curves for the constructed nomogram

**Fig. 5:**
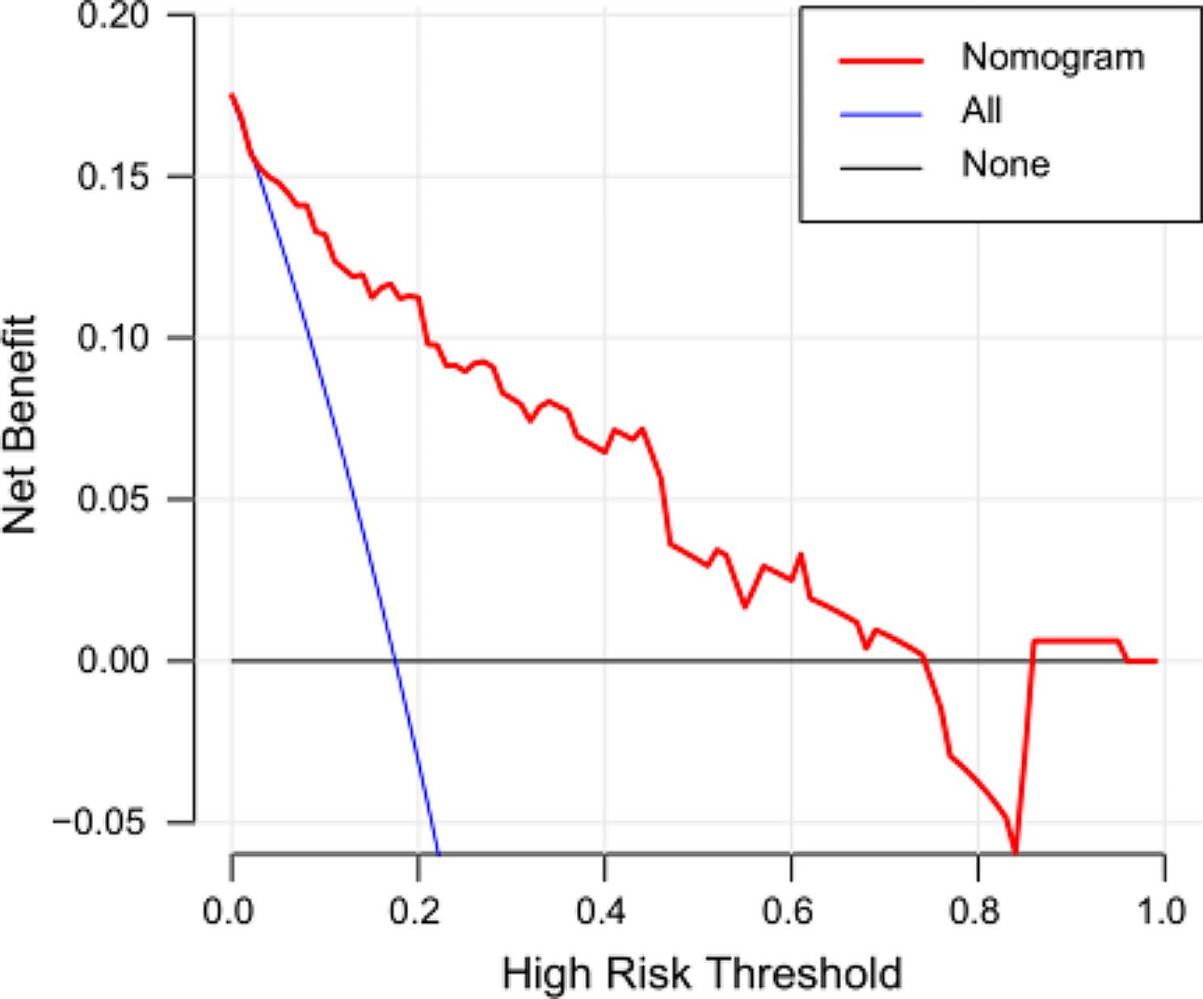
Decision curve analysis for the constructed nomogram evaluating the risk of symptomatic pneumonia

Based on the identified independent risk factors, we developed a nomogram to predict symptomatic pneumonitis in patients with lung cancer receiving thoracic radiation (Fig. 2). The C-index of our nomogram was 0.89 (95% CI: 0.82–0.95), supporting its excellent predictive capacity. Moreover, the ROC curve showed that the AUC value reached 0.89 (95% CI: 0.83–0.95), indicating an excellent distinguishing ability of this model. The comparison of ROC curves between the nomogram and each prognostic factor revealed that the comprehensive model had higher discrimination than any single variable (Fig. 3). Additionally, the calibration curve demonstrated favorable consistency between the predicted symptomatic pneumonitis and the actual observed results, confirming that our nomogram was well calibrated (Fig. 4). DCA displayed the agreeable potential clinical impact of our prediction model (Fig. 5). Furthermore, a risk classification system for symptomatic pneumonitis was generated by recursive partitioning analysis according to the calculated total score of each patient using the nomogram.

### Risk classification system

**Fig. 6:**
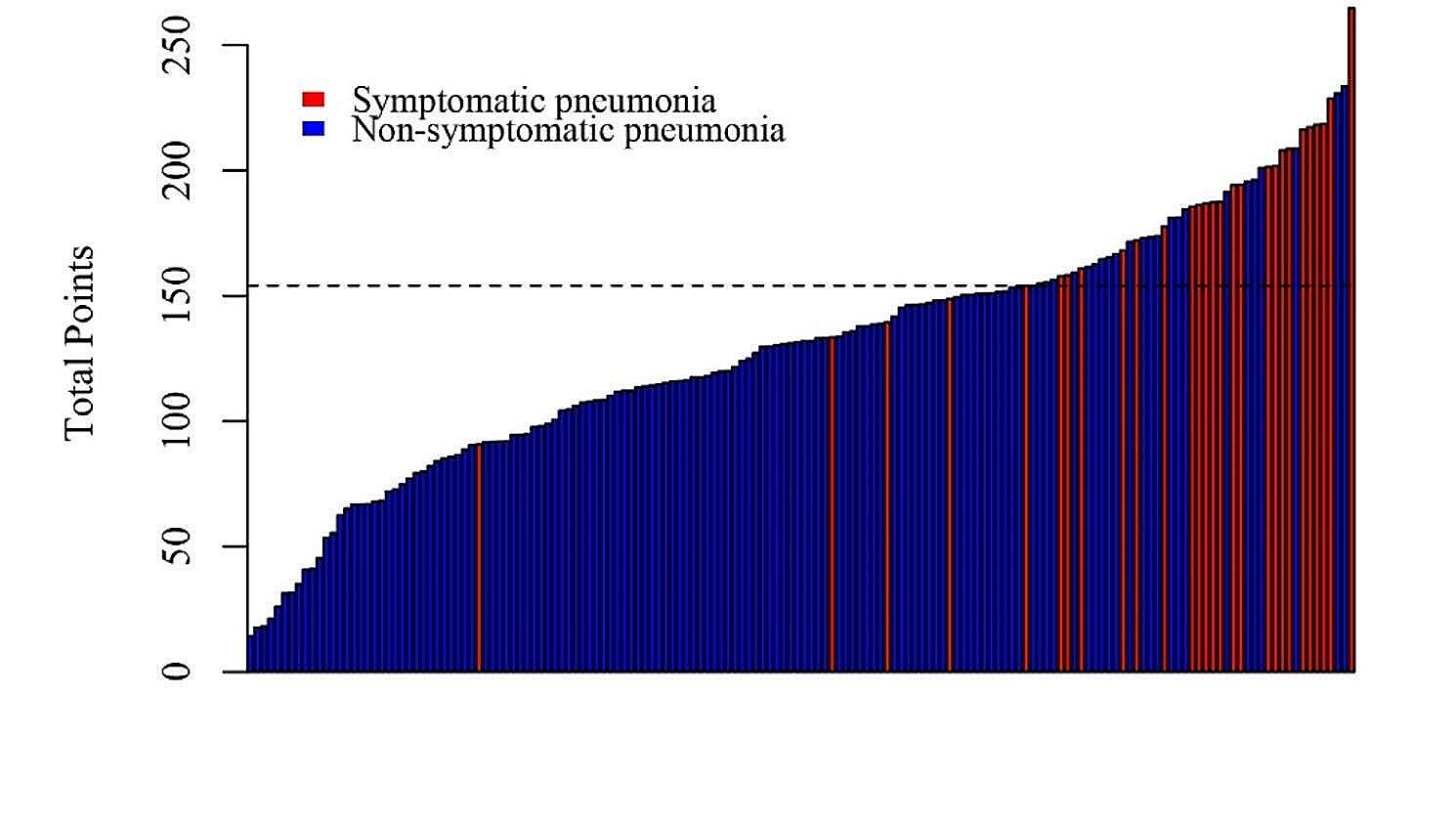
Histogram of nomogram scores for all patients. The red columns represent patients with symptomatic pneumonia., the blue columns represent patients with non-symptomatic pneumonia, and the dotted line indicates the threshold values separating the two risk groups

A novel risk classification system for symptomatic pneumonitis is shown in Fig. 6. The optimal cut-off values were determined as 154. All patients were classified into two risk groups: the low-risk group (score 0-154) and the high-risk group (score > 154). The incidence of symptomatic pneumonitis in the low and high-risk groups was 17.85% (5/28) and 82.14% (23/28), respectively. Further analyses demonstrated that our risk classification system effectively differentiated the incidence risk of RP ≥ 2 risk levels, facilitating decision-making in clinical practice.

### Development of a dynamic web-based calculator

**Fig. 7:**
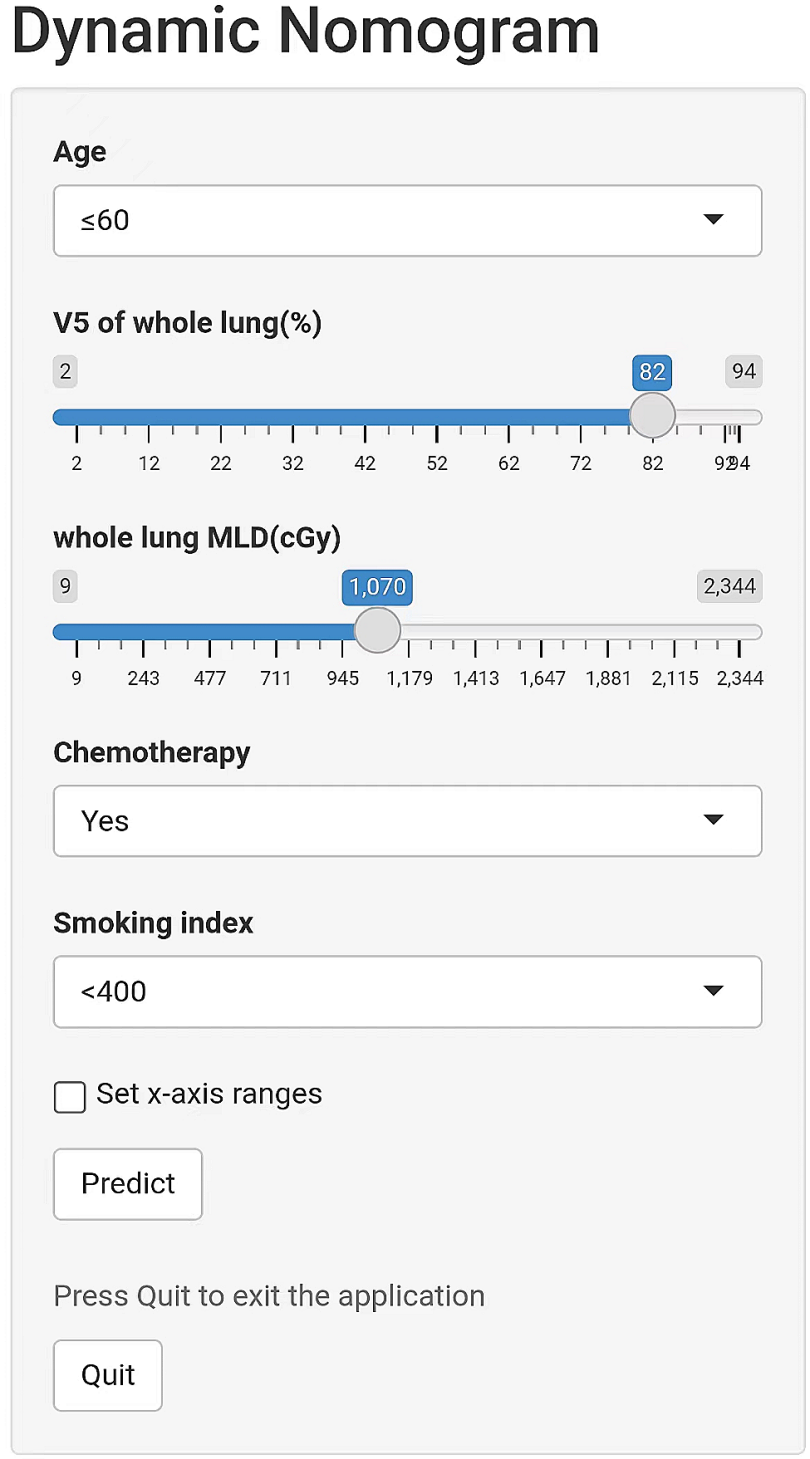
A dynamic web-based survival calculator for predicting the occurrence of symptomatic pneumonia

Based on the established nomogram, we developed a dynamic web-based survival calculator to simplify the application of this nomogram (available at https://rp-2023.shinyapps.io/rp2023/) (Fig. 7). Our web-based calculator for the incidence of symptomatic pneumonitis allows for better identification of patients at high risk for symptomatic pneumonitis and enables timely treatment. By inputting patients’ clinical and dosimetry features, it is convenient to predict the probability of symptomatic pneumonitis.

## Discussion

Radiation pneumonitis (RP) is the most common side effect following radiotherapy for lung cancer. Currently, there is no effective treatment for RP. Symptomatic pneumonitis can impact a patient’s prognosis and reduce their quality of life. It is well-recognized that radiation dose parameters are the most important influencing factors for RP. The lung V20 is a classic and frequently described dosimetry parameter, and limiting lung V20 can significantly reduce the incidence of RP [30]. However, even when the whole lung V20 dose parameter is strictly restricted before radiotherapy, RP is still unavoidable. This suggests that multiple factors, in addition to dose parameter factors, contribute to RP.

Identifying high-risk patients for symptomatic pneumonitis before radiotherapy is crucial to prevent its occurrence. Patients with RP ≥ 2 represent a highly heterogeneous group; our study combined multiple clinical and dosimetry indicators to establish a predictive model. The results demonstrated that a combination of three dosimetry and three clinical factors could predict RP ≥ 2. More importantly, we developed a dynamic nomogram and risk classification system with good predictive performance, which is easy for physicians to understand and implement in practice.

Previous studies have estimated the incidence of symptomatic pneumonitis to be 15–40% among patients undergoing thoracic radiotherapy [14]. Our study’s incidence (17.5%) is consistent with these findings. However, the incidence of fatal pneumonitis in our study was 5% (8/160), significantly higher than the 2% reported in previous studies [8]. In our study, 13 patients had Grade 3 symptomatic pneumonitis, accounting for 46.43% (13/28) of all symptomatic pneumonitis cases. The high mortality rate in our study may be related to the increased number of patients diagnosed with ≥ Grade 3 pneumonitis, which is more likely to develop into fatal pneumonitis.

Older age has been shown to be a predictor of symptomatic pneumonitis [7, 31]. Previous studies have found that older patients (age ≥ 70) are more likely to develop symptomatic pneumonitis [31]. Similar results were confirmed in a prospective study with 7752 patients who received stereotactic body radiation therapy (SBRT) for stage IA to IB non-small-cell lung cancer (NSCLC) [10]. This is primarily due to the fact that older patients have more comorbidities that are a risk factor for RP than younger patients [7]. Although age ≥ 60 years was an independent risk factor in our study, a threshold value has not been determined due to additional risk factors such as smoking status and pulmonary function [3]. While age is an uncontrollable factor, it has minimal impact on the symptomatic pneumonitis score in the nomogram.

The relationship between smoking and radiation-induced lung injury remains controversial [32]. Tobacco smoking is the leading risk factor for developing lung cancer. In contrast, smoking has been found to have a protective effect on the risk of RP. Smokers are less likely to develop radiation pneumonitis, as it is thought that smoking-damaged lungs may be less sensitive to radiation damage [33, 7, 34]. However, smoking is a significantly poor prognostic factor for overall survival [35]. The smoking index, calculated as cigarettes smoked per day × years smoked, was an independent risk factor for symptomatic pneumonitis when the index was ≥ 400 in our study. The relationship between the smoking index and symptomatic pneumonitis has received insufficient attention in lung cancer patients; our results provide solid evidence, but the nomogram showed that the smoking index had the lowest impact among the six risk factors for symptomatic pneumonitis.

Conflicting results exist regarding the effects of concurrent, concomitant, or neoadjuvant chemotherapy on radiation toxicity. A dosimetric analysis of 25 patients with NSCLC revealed that approximately 30% of patients experienced at least a 20% reduction in tumor volume following induction chemotherapy, translating into a slight yet statistically significant reduction (5%) in the predicted risk of RP [36]. However, a retrospective analysis of 223 patients by Wang et al. [37] did not find a significant association in the risk of Grade 3 RP between patients with and without induction chemotherapy. In contrast, another retrospective study of 84 lung cancer patients receiving three-dimensional conformal radiotherapy (3D-CRT) showed that the risk of symptomatic pneumonitis was three-fold higher in the chemotherapy group than in the non-chemotherapy group (31.2%) [38]. Our findings showed that patients who did not receive chemotherapy were more likely to develop symptomatic pneumonia than those who did. We consider the reasons that lead our results to differ from those of previous studies as follows: Some patients in the symptomatic pneumonia group were more prone to symptomatic pneumonia due to older age and poor ECOG score, so we did not give chemotherapy to these patients. In addition, some patients with large lung tumors refuse chemotherapy due to their own wishes, resulting in a larger radiation treatment area, which in turn exposes more normal lung tissue to radiation. Therefore, we believe that the appropriate course of chemotherapy helps to shrink the tumor, and does not increase the occurrence of symptomatic pneumonia.

It should be noted that following the introduction of volumetric modulated arc therapy (VMAT) and SBRT into clinical practice, greater attention has been paid to the potential adverse effects of low-dose lung dose-volume histogram (DVH) parameters on RP ≥ 2. Several studies [12, 29, 39–42] have reported a close relationship between whole lung V5, V10, and RP in lung cancer. Sheng L et al. [40] found that the dose threshold for V5 in patients with non-small cell lung cancer treated with induction chemotherapy was 44%. Another study discovered that ipsilateral lung V5 ≥ 55.65% might be associated with a higher risk of RP [21]. A previous clinical study reported that acute radiation pneumonitis was reduced from 29.2 to 5.7% in lung cancer patients receiving concurrent chemoradiotherapy when V10 ≤ 50 [41]. Moreover, Boonyawan et al. [43] identified a correlation between V10 > 30% and grade ≥ 2 RP in patients who had undergone surgery followed by PORT. Our study found that lung V5 was independent risk factors. We conducted a univariate receiver operating characteristic (ROC) analysis on lung V5, with the best cut-off value being 61.10% (AUC = 0.726, 95% CI: 0.626–0.827, sensitivity = 0.679, specificity = 0.720). The whole lung V5 > 61.1% in the symptomatic pneumonitis group was 67.86% (19/28) compared to 28.03% in the asymptomatic pneumonitis group (37/132). These data differences were statistically significant(*P*<0.05). Our whole lung V5 was mostly above the dose-optimal threshold, which may be associated with higher severity and mortality of symptomatic pneumonitis than previously reported. In conclusion, further research is needed to confirm that our findings are not accidental and to define the optimal cut-offs of whole lung V5 for more accurate prediction of symptomatic pneumonitis.

Mean lung dose (MLD) can be employed to predict the risk of symptomatic pneumonitis in lung cancer patients undergoing radical radiotherapy [44, 45]. A finding concur with these studies, indicating that the incidence of RP ≥ Grade 2 increased in cases where the MLD was ≥ 14.3 Gy [18]. A prior study [21] found that patients with a mean ipsilateral lung dose ≥ 11.86 Gy might be at a higher risk of RP ≥ Grade 2. Another study suggested that MLD of 10 Gy was significantly correlated with an increased risk of RP, while an MLD < 12 Gy was significantly correlated with a low risk of RP ≥ Grade 2 [17]. Lee et al. [46] used perfusion single-photon-emission computed tomography and fluorodeoxyglucose positron-emission tomography imaging to demonstrate that MLD (also functional MLD) was a significant predictor of grade ≥ 2 pneumonitis, with a cutoff value of 13.6 Gy (functional MLD: 13.2 Gy). Baker R et al. [42] found that patients receiving stereotactic body radiation therapy (SBRT) with MLD less than 600 Gy had a lower risk of RP ≥ Grade 2, and the same results were obtained in another study [45]. For patients receiving postoperative radiotherapy, it is recommended to control the whole lung MLD below 10.8 Gy [12]. A machine learning study based on 203 stage II-III lung cancer patients receiving chemoradiotherapy found that whole lung MLD was strongly associated with symptomatic pneumonitis, with a cutoff value of 13.4 Gy [39]. In our study, the best cut-off value of whole lung MLD was 948.15 Gy (AUC = 0.726, 95% CI: 0.622–0.799, sensitivity = 1.00, specificity = 0.424), indicating that the incidence of symptomatic pneumonitis was definite in cases where the whole lung MLD was > 948.15 Gy. Although the cutoff values for MLD differ across research institutions, our findings were within the range previously reported.

As is well-known, multifactorial combined prediction models are beneficial in enhancing RP prediction performance. Although several prediction models have been preliminarily established in prior studies, their implementation in the clinic has been limited due to their unsatisfactory ability to identify RP or the practical challenges they pose. In our study, however, a number of essential variables, including age, smoking index, whole lung V5 and whole lung MLD, were integrated into a nomogram to accurately personalize the risk assessment of RP ≥ 2 for each patient. The c-index of the nomogram in this study was 0.89, indicating that it outperformed previously reported models combining clinical dosimetric factors in predicting RP ≥ 2. The transformation into a dynamic nomogram makes it more convenient for clinicians to apply. More importantly, a new risk classification system was established that effectively stratified lung cancer patients into distinct RP risk groups. Our study demonstrates that the model exhibits excellent discrimination in predicting the occurrence of RP ≥ 2. As any physician can easily measure the included variables, the practicability of the model is enhanced. Despite the many advantages of our study, some limitations should be acknowledged. First, Due to the small sample size, we did not set up a validation group. Although there is no external validation, our concordance index and calibration plots confirmed the good predictive performance of our model. This study is a preliminary study, we will continue to collect more patients and expand the sample size to verify the accuracy of our nomogram. Second, as this study is retrospective and from a single institution, there may be a selection bias. In addition, our previous clinical data are incomplete, lacking crucial information such as inflammatory factors and specific chemotherapy courses, which may help improve the accuracy of symptomatic pneumonitis prediction. Lastly, only symptomatic pneumonitis was used as an endpoint, while local tumor control and overall survival were not considered. Reducing the risk of symptomatic pneumonitis may concurrently increase the risk of local tumor control failure and alter patient survival time. In the future, multi-objective models will be developed to assist physicians in determining the best course of treatment. This study only employed symptomatic pneumonitis as an endpoint and did not consider other treatment outcomes, such as local tumor control and overall survival rate. Reducing the risk of RP ≥ 2 may simultaneously increase the risk of tumor local control failure and decrease survival time; thus, our prospective study is ongoing.

## Conclusion

We developed a dynamic nomogram and risk classification system by incorporating age, smoking index, chemotherapy, whole lung V5 and whole lung MLD. Our results demonstrated that the model performs well in helping physicians recognize high-risk RP ≥ 2 lung cancer cases and guide individualized treatment and clinical decision-making.

## Data Availability

All data acquisition from the medical record management system of Zhongshan People’s Hospital. The datasets used and/or analysed during the current study are available from the corresponding author on reasonable request.

## Abbreviations

3D-CRT: Three-dimensional conformal radiotherapy
AUC: Area under the curve
BED: Biological effective dose
C-index: Concordance index
CTCAE, 5.0: The common adverse reaction event evaluation criteria
DCA: Decision curve analysis
DVH: Dose-volume histogram
DVH: Dose-volume histogram
GTV-nd: Gross lymph node volume
GTV: Gross tumor volume
ICIs: Immune checkpoint inhibitors
IMRT: Intensity-modulated radiotherapy
MLD: Mean lung dose
NSCLC: Non-small-cell lung cancer
NTCP: Normal tissue complication probability
PORT: Postoperative radiotherapy
PTV: Planning target volume
ROC: Receiver operating characteristic
RP ≥ 2: Symptomatic pneumonitis
RP: Radiation pneumonitis
SBRT: Stereotactic body radiation therapy
VMAT: Volumetric modulated arc therapy
Vx: percentage of whole lungs volume receiving x Gy
